# Distribution patterns of human papillomavirus genotypes among women in Guangzhou, China

**DOI:** 10.1186/s13027-023-00541-8

**Published:** 2023-10-31

**Authors:** Shu Li, Kelan Zhang, Liu Yang, Jia Wu, Neha Bhargava, Yinghua Li, Fei Gao

**Affiliations:** 1 Department of Clinical Laboratory, Guangzhou Women and Children’s Medical Center, Guangzhou Medical University, No. 9 Jinsui Road, Tianhe District, Guangzhou, 510620 Guangdong China; 2 Reproductive Medicine Center, Tongji Hospital, Tongji Medical College, Huazhong University of Science and Technology, No.1095 Jie Fang Avenue, Wuhan, 430030 Hubei Province China; 3 Department of Medicine and Surgery, Guangzhou Medical University, Xinzao Road, Guangzhou, 511436 Guangdong China; 4 Center Laboratory, Guangzhou Women and Children’s Medical Center, Guangzhou Medical University, 318 Renminzhong Road, Yuexiu District, Guangzhou, 510120 China

**Keywords:** HPV infection, Genotype distribution, Guangzhou

## Abstract

**Background:**

Cervical cancer is associated with high‐risk human papillomavirus (HR-HPV) infection in the world. We aimed to evaluate the status of HPV infection among women in Guangzhou, China.

**Methods:**

The study recruited 28,643 female patients from the Guangzhou Women and Children’s Medical Center for HPV genotype testing between 2019 and 2021.

**Results:**

5668 patients were infected with HPV, resulting in an overall infection prevalence of 19.78%. The prevalence of HR-HPV was recorded at 13.94% (both single-infections and multi-infections), probably high-risk HPV/possibly carcinogenic (pHR-HPV) as 3.51%; and low-risk HPV (LR-HPV) as 3.56%. The most common HR-HPV genotype detected was HPV-52 with an infection rate of 4.99%, followed by HPV 58 (2.18%), 16 (2.12%), 51 (1.61%), 39 (1.19%), 56 (1.09%), 59 (0.85%), 18 (0.72%), 33 (0.61%), 31 (0.53%), 35 (0.20%), 45 (0.17%). Among LR-HPV genotypes, HPV-42 was the most common (1.08%), followed by 44 (0.77%), 81 (0.68%), 6 (0.48%), 43 (0.40%), 11 (0.23%) and 83 (0.07%). The prevalence of infection among different genotypes in pHR-HPV was: 68 (1.29%), 53 (1.21%), 66 (0.77%), 82 (0.25%), 73 (0.16%). Additionally, the prevalence of single genotype HPV infection exceeded that of multiple HPV infections except HPV-59.

**Conclusion:**

Our findings imply that HPV genotype infections in Guangzhou demonstrate a regional and age-related distribution. Therefore, these data can provide a substantial foundation for further epidemiologic analysis to control and prevent HPV infections in Guangzhou.

**Supplementary Information:**

The online version contains supplementary material available at 10.1186/s13027-023-00541-8.

## Introduction

Cervical cancer (CC) stands as the fourth most common cancer and a leading cause of cancer-related deaths among women worldwide, accounting for an estimated 604,000 new cases and 342,000 deaths in 2020 [1]. Owing to its large population, China accounts for 11.9% of global CC deaths [2]. Numerous studies have shown a strong association between several types of human papillomavirus (HPV) and cervical precancerous lesions [3]. It is recognized that CC caused are attributed to high-risk HPV types (HR-HPV) in all body regions [4]. Twelve HR-HPVs have been identified as cancer-causing and classified as Group 1 carcinogens in the International Agency for Research on Cancer monograph. In addition, HPV infection is implicated in the majority of CC [5], head and neck [6], anal and vulvar cancers, as well as other cancer types.

Over 200 types of HPV have been identified, with these genotypes categorized based on their risk for causing CC into: HR-HPV, low-risk HPV (LR-HPV), and probably high-risk HPV/possibly carcinogenic (IARC Groups 2A and 2B, respectively), hereafter referred to as pHR-HPV [7, 8]. HR-HPV types encompass HPV-16, 18, 31, 33, 35, 39, 45, 51, 52, 58, 56, and 59 [4]. The LR-HPV types comprise HPV-6, 11, 28, 32, 40, 42, 43, 44, 54, 55, 57, 61, 62, 71, 72, 74, 81, 83, 84, 86, 87, and 89 etc. [8]. Lastly, the probably high-risk types encompass HPV-68, while the possibly carcinogenic types include HPV-26, 53, 66, 67, 70, 73, and 82 [4, 9].

Several studies have affirmed that persistent HR-HPV infection is the primary risk factor for CC [10]. In addition, different HPV genotypes exhibit distinct oncogenic capabilities, and certain types of HR-HPV prevail in specific geographic regions [11]. In North America (Canada and the United States), HPV-16 is the most common genotype found in low-grade squamous intraepithelial lesions (LSILs), accounting for 26.3% of a study sample of 2425 patients with an average age of 47.8 (± 12.9) years [12]. On the other hand, Patients from Africa aged 33.9 years (± 11.4 years) infected with HPV-16 were twice as many as those from southern Europe aged 56.5 years (± 14.3 years) [12]. Additionally, HPV prevalence in China was 84.37% in a meta-analysis of 2950 cervical intraepithelial neoplasia (CIN)1 patients and 5393 CIN2/3 patients, but the distribution of HPV types varies across regions [13]. In western China (Tibet Autonomous Region, Chongqing, Guizhou, Shaanxi), HPV-52, 16, 58, and 53 were the most commonly detected HR-HPV types, with the highest HPV detection rate observed in the 36–50 age group [14]. In Northeast China, the predominant HPV types identified in a survey of 110,927 women aged 18–80 years were HPV-16, 58, 52, 33, 53, and 18 [15]. In Beijing, a survey of 46,365 sexually active women between 2017 and 2020 found that the most frequently detected HR-HPV types were HPV-52, 58, 16, 51, 66, and 59 [16]. Several regions in central China, such as Zhengzhou (healthy women aged 25–64 years) [17], Wuhan (patients including physical examination, infertility and vaginitis, and the subjects were aged between 16 and 83 years) [18], and Sichuan (healthy women aged 15–94 years), had the high prevalence of HPV-52 infection [19]. Therefore, it is useful to understand the distribution of HPV types in the population of a region to guide the application of HPV vaccine and CC screening strategies in prevention efforts.

This study focused on the epidemiology of HPV infection in Guangzhou in the past 3 years from January 2019 to December 2021. It is hoped that the study results will provide effective strategies for HPV prevention in Guangzhou and promote HPV-targeted vaccination by the local government.

## Materials and methods

### Study population

The study involved 28,643 women who underwent annual routine gynecological examinations at the outpatient gynecological clinic of Guangzhou Women and Children’s Medical Center between January 2019 and December 2021. Patients’ etiologies consisted of cervical cytological abnormalities like atypical squamous cells and cervical intraepithelial neoplasia (CIN) 1/2/3 also detected during histological examination. It also includes patients with genital warts, physical infertility, vaginitis, cervicitis.

### Ethical standards

The clinical protocol of this study was approved by the Clinical Research Committee of Guangzhou Women and Children’s Medical Center. All participants signed a written informed consent form saying that all procedures will be performed according to the standard. The authors assert that all research procedures comply with ethical standards set by relevant national and institutional human experimental committees, as well as the 1975 Helsinki Declaration, updated by the World Medical Association in 2008.

### Specimen collection and management

On the day of sample collection, participants were required not to be menstruating, not to have received any pelvic or vaginal treatments, and to abstain from sexual activity in the previous 24 h. To obtain the cervical sample, a cotton swab was utilized to delicately remove excess secretions from the cervical orifice. The cytobrush was inserted into the cervical opening at a depth of 1–1.5 cm until the outermost bristles of the brush touched the ectocervix, and gently rotated five times to collect a sufficient number of exfoliated cells. The samples were placed in the preservation fluid bottle for the specimen and stirred several times to remove as many cells as possible. The sample is preserved in a solution maintained at − 80 °C until it is ready for extraction and genotyping of HPV DNA. The collected specimens can be kept at room temperature for no more than 1 week and at 4 °C for no more than 2 weeks. HPV sample genotyping is completed within 1 week.

### Test method

Genomic DNA was extracted from collected samples using a DNA extraction kit (TIANGEN, DP304, Beijing, China) according to the manufacturer’s instructions. All DNA samples were amplified using Cobas Z 480 Real-Time PCR and its corresponding reagents (HybriBio Ltd, HBRT-H13C, Chaozhou, China). HPV genotyping was performed using the HybriMax HPV Gene Array Assay Kit (HybriBio Ltd, C230503A, Chaozhou, China) according to the manufacturer’s instructions. Positive and negative controls were included in each PCR assay to ensure accuracy and check for possible contaminations. This method was utilized to distinguish 23 HPV types, including 16-HR-HPV types. The infection rates among individuals who tested positive for HR-HPV were as follows: 52, 58, 16, 51, 39, 56, 59, 18, 33, 31, 35, 45. Prevalence of LR-HPV infections were as follows: 42, 44, 81, 6, 43, 11 and 83. The prevalence of pHR-HPV-positive infections were 68, 53, 66, 82, 73. In the final step of the process, the different genotypes are identified by adding the Nitro Blue Tetrazolium/5-Bromo-4-chloro-3-indolyl-phosphate (NBT/BCIP) solution to show results, where clearly visible indigo dots indicate HPV positivity. The placement of the HPV genotype probe on the microarray chip determines the outcome, with multiple marks indicating the presence of co-infection or multiple HPV infections.

### Data analysis

All data was analyzed using the SPSS 16.0 software package. Participants’ age and other measurements were expressed as mean ± standard deviation (SD). Chi-square tests were used to compare HPV prevalence in each group. The comparison of data between different ages was considered statistically significant at *P* < 0.0017.

## Results

### The overall prevalence of HPV infection

**Fig. 1:**
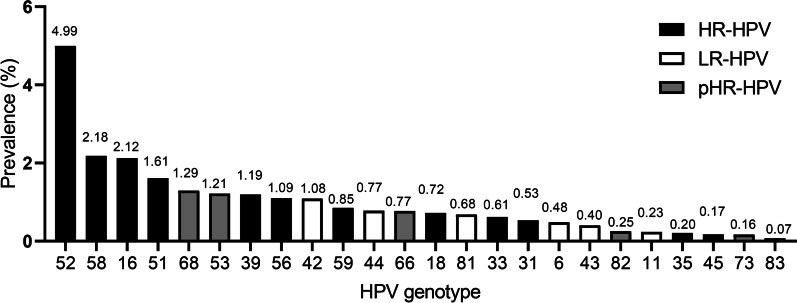
**Distribution of HPV infection rates for different types.** Black represents the prevalence of high-risk HPV (HR-HPV) genotypes; white represents the prevalence of low-risk HPV (LR-HPV) genotypes; grey represents the prevalence of probably high-risk HPV/possibly carcinogenic HPV (pHR-HPV) genotypes

Out of the 28,643 participants, their ages ranged from 16 to 83 years (with an average age of 36.69 ± 8.72), and they were divided into eight groups, each spanning 5 years. The main reasons for HPV testing in these participants included Cervical intraepithelial neoplasia (CIN)1/2/3 (14.78%), biopsy pathology with genital warts (0.03%), physiologic infertility (17.37%), vaginitis (30.61%), cervicitis (37.21%) (Additional file 1: Table S1). The largest number of HPV infections in CIN 1/2/3, physiological infertility, vaginitis and cervicitis were predominantly in the 31–35 age (Additional file 1: Table S2). In addition, there were more HR-HPV infections in each disease than in the pHR-HPV and LR-HPV groups (Additional file 1: Table S3). The results showed that 3992 (13.94%) were HR-HPV, 1021 (3.56%) were LR-HPV, and 1005 (3.51%) were pHR-HPV, including co-infection. The number of positive HR-HPV infections was higher than that of LR-HPV infections. The infection rates for HR-HPV were as follows: 52 (4.99%), 58 (2.18%), 16 (2.12%), 51 (1.61%), 39 (1.19%), 56 (1.09%), 59 (0.85%), 18 (0.72%), 33 (0.61%), 31 (0.53%), 35 (0.20%), 45 (0.17%). Prevalence of LR-HPV infections were as follows: 42 (1.08%), 44 (0.77%), 81 (0.68%), 6 (0.48%), 43 (0.40%), 11 (0.23%) and 83 (0.07%). The prevalence of pHR-HPV-positive infections were 68 (1.29%), 53 (1.21%), 66 (0.77%), 82 (0.25%), 73 (0.16%) (Fig. 1 and Additional file 1: Table S4).

**Fig. 2:**
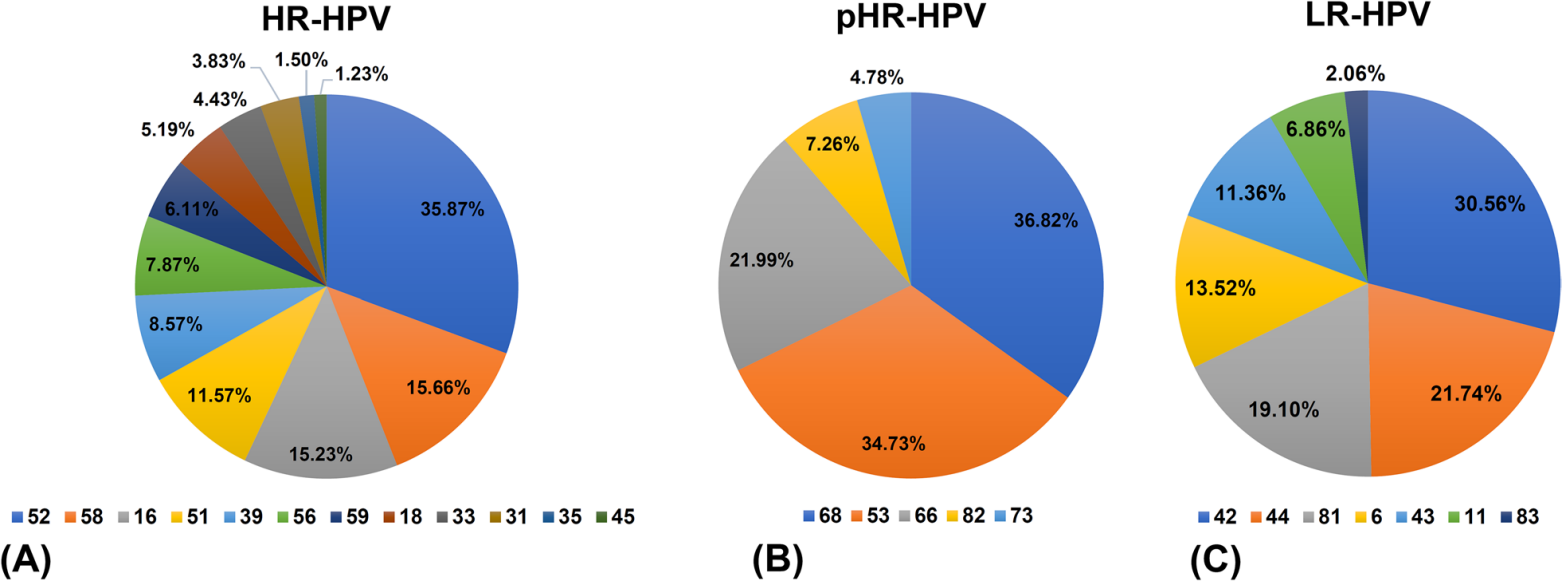
**Relative prevalence of different genotypes by carcinogenic risk. A** Prevalence of each of the HR-HPV genotypes compared to total HR-HPV infections. **B** Prevalence of each of the pHR-HPV genotypes compared to total pHR-HPV infections. **C** Prevalence of each of the LR-HPV genotypes compared to total LR-HPV infections

Single and multiple infections with different HPV genotypes in HPV-positive individuals. Co-infections include multiple HPV genotypes, such as HR-HPV with HR-HPV, HR-HPV with LR-HPV, pHR-HPV and LR-HPV, etc. Among the 5668 patients identified with HPV infection (including single and multiple infections), there were 3992 cases of HR-HPV infections, 1021 cases involving LR-HPV, and 1005 cases of pHR-HPV infections. The types of HR-HPV infections were: HPV-52, 58, 16, 51, 39, 56, 59, 18, 33, 31, 35 and 45. The numbers and percentages of these infections were 1,432 (35.87%), 625 (15.66%), 608 (15.23%), 462 (11.57%), 342 (8.57%), 314 (7.87%), 244 (6.11%), 207 (5.19%), 177 (4.43%), 153 (3.83%), 60 (1.50%) and 49 (1.23%), respectively (Fig. 2A and Additional file 1: Table S5). The types of pHR-HPV infections were: 68, 53, 66, 82 and 73. The numbers and percentages of these infections were: 370 (36.82%), 349 (34.73%), 221 (21.99%), 73 (7.26%), 48 (4.78%) (Fig. 2B and Additional file 1: Table S5). The different types of LR-HPV positive infections identified were HPV-42, 44, 81, 6, 43, 11, and 83. The numbers and percentages of these infections were: 312 (30.56%), 222 (21.74%), 195 (19.10%), 138 (13.52%), 116 (11.36%), 70 (6.86%) and 21 (2.06%) of cases, respectively (Fig. 2C and Additional file 1: Table S5). Analysis of data on the proportion of different HPV types in the patient group that were positive for the virus revealed HPV-52 as the most prevalent subtype. Following HPV-52 were the HR-HPV types 58, 16, 51. Among the LR-HPV types, HPV-42 was the most common, followed by HPV-44, 81, and 6 (Fig. 2C and Additional file 1: Table S5).

### Age distribution features of HPV infection

Upon data analysis, we discovered that the average age of individuals with HPV in Guangzhou was 37.05 ± 10.02 years. We divided the patients into eight age groups, and the infection rates for these groups were as follows: 24.02% (482/2,007) for those ≤ 25 years old, 19.31% (1109/5742) for individuals aged 26–30 years, 18.96% (1343/7082) for those aged 31–35 years, 18.74% (989/5278) for individuals aged 36–40 years, 19.08% (603/3160) for those aged 41–45 years, 18.46% (459/2487) for individuals aged 46–50 years, 20.81% (304/1461) for those aged 51–55 years, and 26.58% (379/1426) for those ≥ 56 years old (Additional file 1: Table S6). In comparison to the ≤ 25 years age group, the ≥ 56 years age group showed no significant difference, but exhibited a higher HPV infection rate than all other age groups, including the 26–30 years, 31–35 years, 36–40 years, 41–45 years, 46–50 years, and 51–55 years groups (Additional file 1: Table S7). The above findings indicate that individuals in the ≥ 56 years age group had a higher HPV-52 infection rate (*P* < 0.0017) compared to other age groups, except for the ≤ 25 years age group (*P* = 0.234) and the 51–55 years age group (*P* = 0.026) (Fig. 4, and Additional file 1: Tables S8 and S9).

**Fig. 3:**
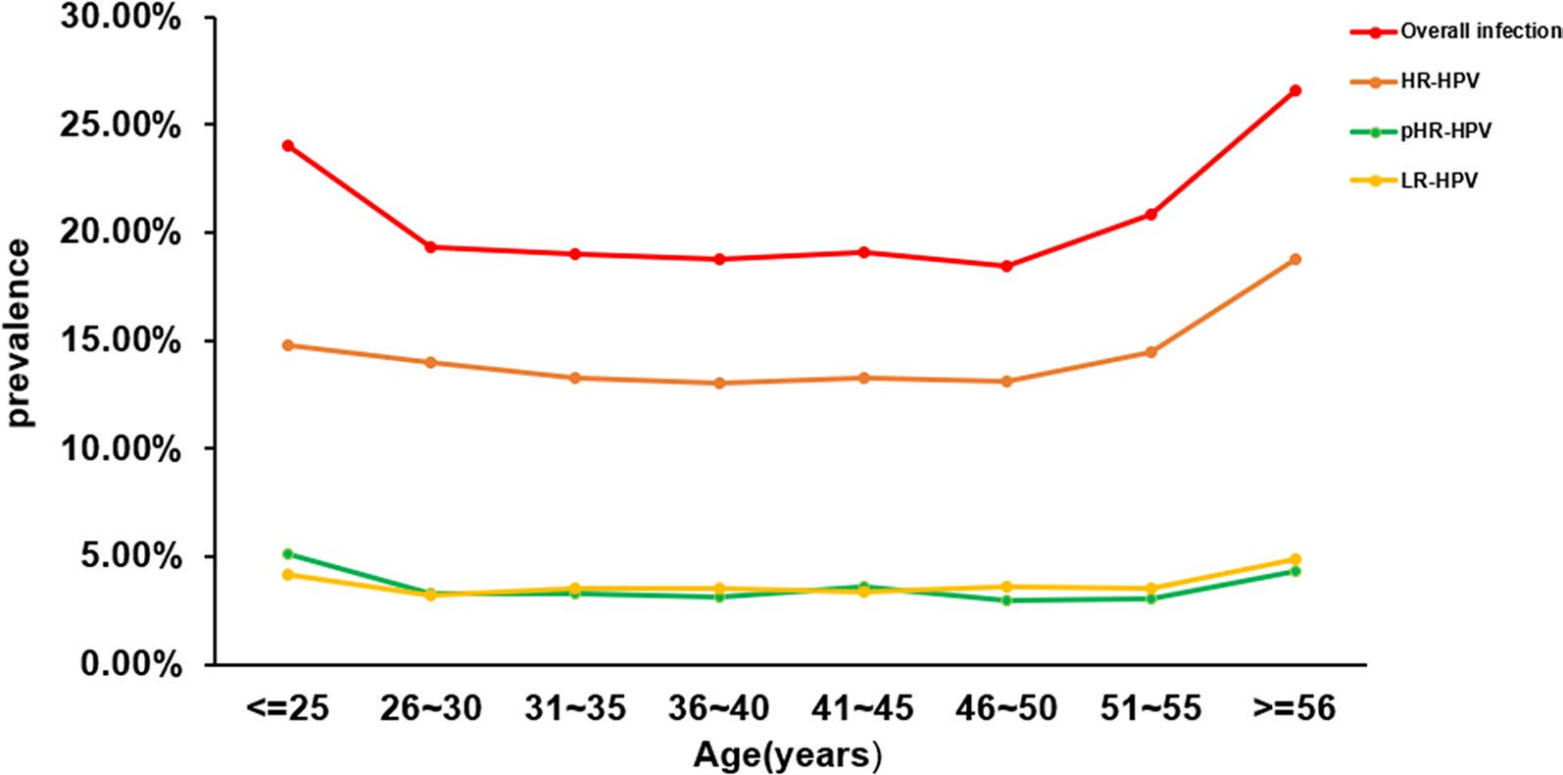
**Prevalence rates of HPV infections by age.** HR-HPV, pHR-HPV, and LR-HPV infections correspond to the rate of positivity among total HPV genotypes, respectively

Based on an analysis of trends in the age group affected by HPV infections, both the total infection rate and HR-HPV declined after peaking at age 25 or younger, and continued to decline until age 45. However, after age 46, there was an increase in the prevalence rate (Fig. 3). Between the ages of 46 and 56, the overall infection rate continued to increase, along with the presence of HR-HPV infections (Fig. 3). The prevalence of LR-HPV infection declined after age 25 and remained relatively low until age 55. However, prevalence then began to rise again after age 55 (Fig. 3).

**Fig. 4:**
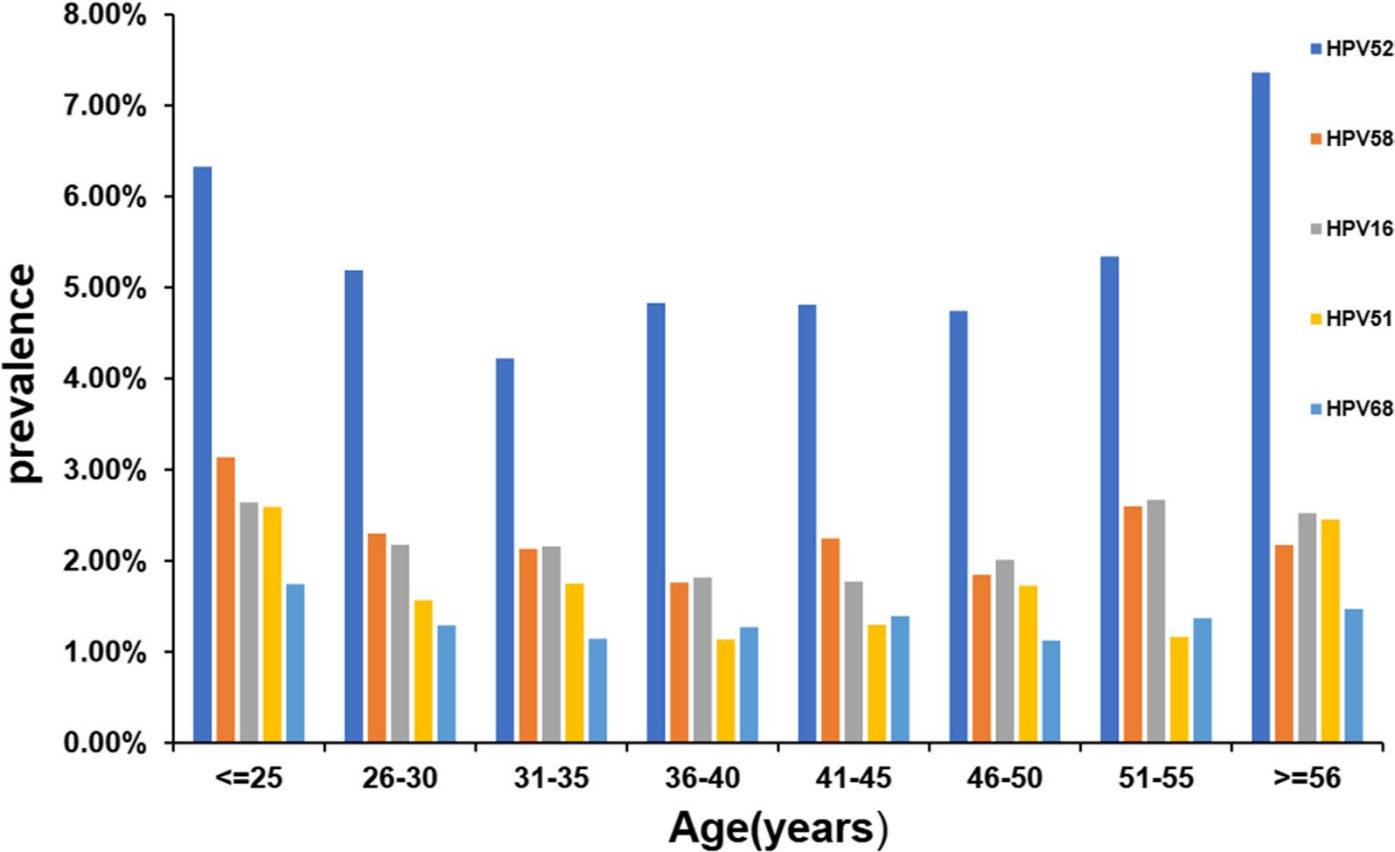
**Age-specific prevalence of the top 5 most frequently occurring HPV genotypes. **Blue, orange, grey, yellow and sky blue represent the prevalence of HPV-52, 58, 16, 51 and 68 genotypes in different age groups, respectively

After studying the occurrence of HR-HPV, LR-HPV, and overall HPV infection in women of different age groups, we noted a decrease in the prevalence of HPV infection in middle-aged women. However, the prevalence of LR-HPV infection showed no significant change across the age groups we observed (Fig. 3 and Additional file 1: Table S5). The prevalence of the five most common HR-HPV genotypes in each age group indicates that the prevalence of HPV-52 infection increases with age from 31 to 56 (Fig. 4). Furthermore, the prevalence of HPV-52 infection was higher in the 51–55 age group than in any other age group, except for the ≥ 56 age group and the ≤ 25 age group (Additional file 1: Table S9).

### Comparison of the number and genotype of HPV single and multiple infections

**Fig. 5:**
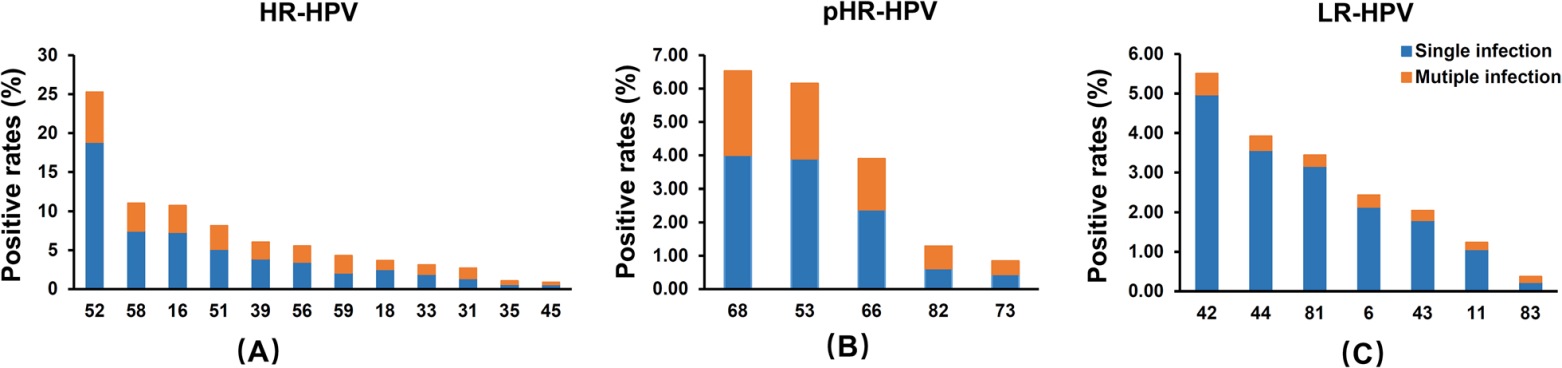
**Distribution of single and multiple infections of HR-HPV, pHR-HPV and LR-HPV genotypes.** **A** Prevalence of single and multiple infections by HR-HPV genotype. **B** Prevalence of single and multiple infections by pHR-HPV genotype. **C** Prevalence of single and multiple infections by LR-HPV genotype

The analysis of infection numbers for different HR-HPV genotypes showed that the number of single infections was significantly higher than that of multiple genotypes, except for HPV 59 (Fig. 5A, Table 1 and Additional file 1: Table S10). For pHR-HPV, the number of monogenic infections, including 68, 53, 66, and 73, was also significantly higher than multiple infections except for HPV-82 (Fig. 5B, Table 1 and Additional file 1: Table S10). As for LR-HPV, the number of single infections, including 42, 44, 81, 43, 6, 11, and 83, was significantly higher compared to multi-genotype infections (Fig. 5C, Table 1 and Additional file 1: Table S10).

**Table 1:** Numbers and rates of single and multiple HPV infections

Genotype/genotypes	Single	Multiple (2)	Multiple (3)	Multiple (4)	Multiple (5)	Multiple (6)
Positive number	4740	760	132	26	7	3
Constituent ratio	83.63%	13.41%	2.33%	0.46%	0.12%	0.05%

### Four HR-HPV genotypes exhibit a wide distribution of prevalence in less economically developed regions of China

**Fig. 6:**
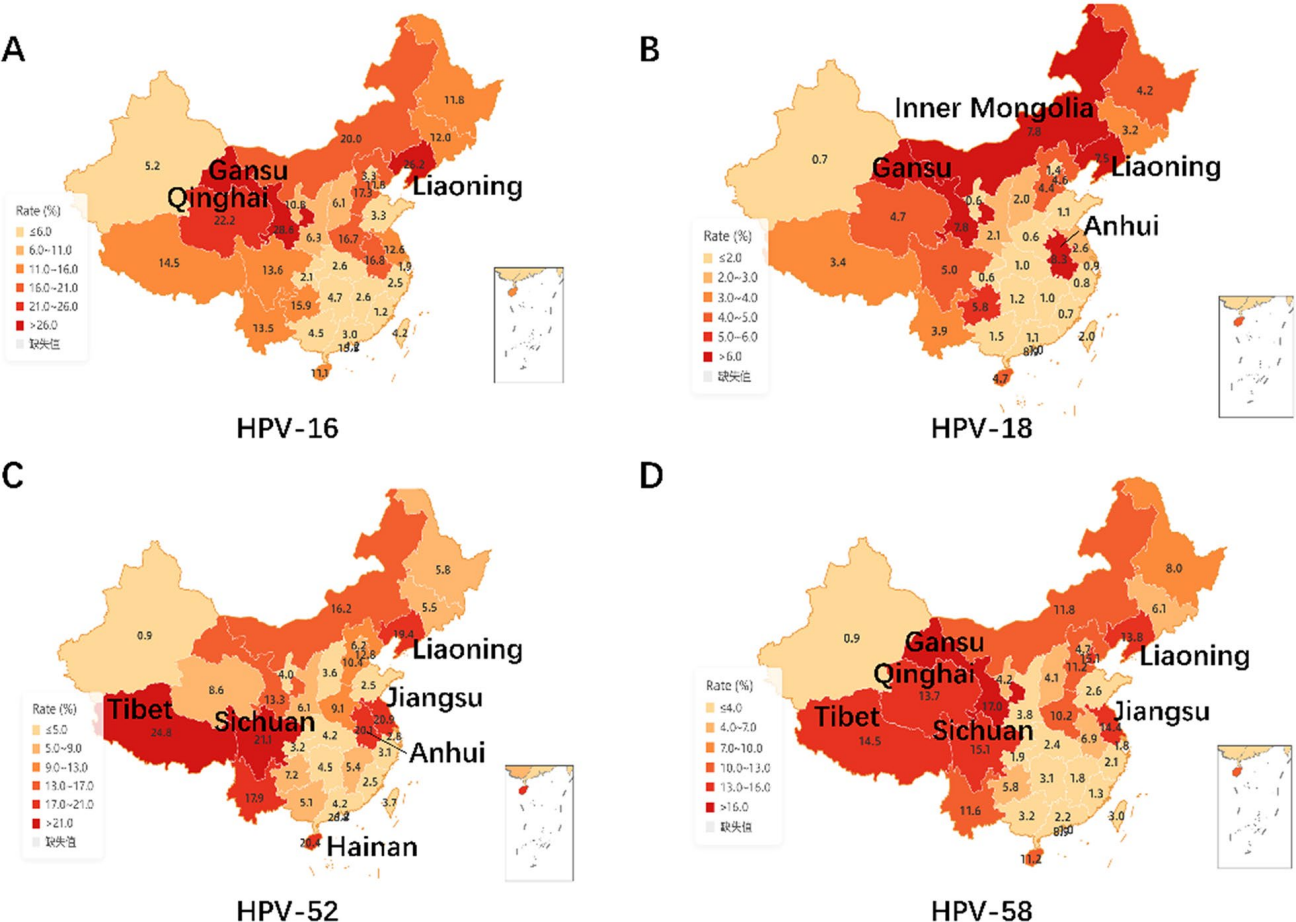
**Prevalence distribution of four HR-HPV genotypes in China.** **A** Distribution of HPV-16 genotype infection rates in different regions of China. **B** Distribution of HPV-18 genotype infection rates in different regions of China. **C** Distribution of HPV-52 genotype infection rates in different regions of China. **D** Distribution of HPV-58 genotype infection rates in different regions of China. Darker colours indicate higher infection rates

As the HPV genotypes distribution in China is so peculiar, we summarized the epidemiological distribution of the four common HR-HPV genotypes (HPV-16, 18, 52, 58) in China by referring to the studies of HPV prevalence in all regions of China over the past 10 years (Additional file 1: Table S11). The results showed that the prevalence of HPV-16 genotypes was higher in Gansu (28.59%), Liaoning (26.20%), and Qinghai (22.21%) than in other provinces and cities (Fig. 6A); and the prevalence of HPV-18 genotypes was higher in Macau (8.90%), Anhui (8.26%), Inner Mongolia (7.76%), Gansu (7.77%), and Liaoning (7.50%) provinces (Fig. 6B). The prevalence of HPV-52 genotype was higher in Tibet (24.80%), Sichuan (21.05%), Jiangsu (20.93%), Hainan (20.40%), Anhui (20.07%), and Liaoning (19.40%) than in other provinces (Fig. 6C). In addition, the prevalence of HPV-58 genotype was higher in Gansu (17.01%), Sichuan (15.14%), Tibet (14.50%), Jiangsu (14.43%), Liaoning (13.80%), and Qinghai (13.74%) than in other regions (Fig. 6D). These indicate that the prevalence of these four HR-HPV is mainly in some economically underdeveloped regions of China. Comparison of this investigation of ours shows that Guangzhou, as the center city of Guangdong province, is one of the more economically developed cities in China, the prevalence of these four HPV genotypes is lower, and close to the same as the results of other Guangdong Province studies.

## Discussion

According to the study, data show that 5668 individuals in Guangzhou were diagnosed with HPV in the past 3 years, resulting in an overall infection rate of 19.78%. The predominant genotype for HR-HPV infection was HPV-52, followed by HPV 58, 16, 51, 39, and 56. For LR-HPV, HPV-42 was the most common genotype, followed by HPV-44, 81, and 6. We also conducted an analysis of the age groups among the infected patients. The highest prevalence was observed in patients aged 56 years and older, with the second-highest prevalence recorded among patients under 25 years of age. This finding raises concerns as it suggests a significant infection rate in both these age groups. Women ages ≥ 56 have higher rates of HPV infection, which may be due to their immune system being less efficient at eliminating HPV infection, as well as their sex hormone status or vaginal epithelial functioning [20]. This data could serve as a foundation for further epidemiological analysis of HPV infection control and prevention in the region.

Persistent HR-HPV infection has a strong association with the development of CC in women [21]. Although HPV vaccines have been available in many countries since 2007, there has been no vaccine development that considers the distribution and prevalence of different HPV genotypes [21]. The July 2020 WHO report and subsequent studies reported that global coverage of HPV immunization stands at just 15% [22–24]. It is estimated that cumulative HPV vaccine coverage for women aged 9–45 in China between 2018 and 2020 is only 2.24% [25]. Therefore, there is still a long way to go to ensure universal access to HPV vaccines.

In this study, the age of participants was primarily concentrated between 16 and 83 years, with a mean age of HPV-infected patients at 37.05 ± 10.02 years. Numerous studies have demonstrated a strong association between HPV infection and sexual activity [21]. Furthermore, previous analyses have indicated that HPV infection can be acquired relatively soon after the onset of sexual activity. Therefore, it is of utmost importance to engage in HPV vaccination prior to initiating sexual activity in order to mitigate the risk of HPV infection to some extent.

Data from the current study showed that HPV infection was mainly concentrated in the under-25 and over-56 age groups, with the highest prevalence of HPV-52 in the under-25 and over-56 age groups (Fig. 4), at 6.33% and 7.36%, respectively. The total HPV infection rates, HR-HPV infection rates, and LR-HPV infection rates in the over-56 age group (respectively 26.58%, 18.72% and 4.91%) were the highest among all age groups, which may be due to the effects of menopause or ovarian function decline.

We conducted a comparison between two studies on the prevalence of HPV genotypes in Guangzhou. The first study, conducted in 2011, involved a sample of 250 healthy women aged 20–63 years [26]. This study, conducted at the Obstetrics and Gynecology Clinic of the Second Affiliated Hospital of Sun Yat-sen University in Guangzhou from January 2007 to April 2008, revealed that HPV-52 (2.62%) was the most common high-risk type among the Guangzhou population. The highest prevalence of HPV infection was observed in the age group of 20–29 years. The second study, conducted in 2022 [27], sampled a total of 6480 patients from the gynecology outpatient clinic of the Guangzhou Women and Children’s Medical Center. This study, carried out from August 2020 to September 2021, included healthy individuals as well as those with other cervical diseases. The results showed that HPV-52 infection was the most prevalent at 5%, followed by HPV-16 (2.3%), HPV-58 (1.8%), HPV-39 (1.6%), and HPV-51 (1.5%). The highest prevalence of HPV infection was found in the age group ≤ 24 years. Our own study, covering a period of nearly 3 years from January 2019 to December 2021, utilized a sample from the Guangzhou Women and Children’s Medical Center. Our findings also indicated the highest prevalence of HPV-52 at 4.99%. In comparison to the previous two studies, our results were similar but had a higher rate due to the larger sample size and wider range of study subjects. The prevalence rates of HPV-58 (2.18%) and HPV-51 (1.61%) were higher than those of HPV-16 (2.12%) and HPV-39 (1.19%), respectively. However, overall, HR-HPV infections were still predominantly dominated by these genotypes, and the rates were not significantly different.

HPV vaccination for women of the right age is an important measure to effectively prevent and control HPV infection and thus reduce the incidence of cervical cancer. From 2016 until May 2022, five HPV vaccines have been approved for registration in China, including three imported HPV vaccines and two domestic vaccines. They are: bivalent vaccine from Vantage Cang hai Biotech (China) and GlaxoSmithKline, Merck Sharp & Dohme quadrivalent vaccine and nine-valent vaccine. As we all know, bivalent vaccine mainly targets HPV-16, 18 genotypes; quadrivalent vaccine mainly prevents HPV-6, 11, 16, 18 genotypes; and nine-valent vaccine is mainly used to prevent HPV-6, 11, 16, 18, 31, 33, 45, 52, 58 genotypes. From 2018 to 2020, the number of HPV vaccinations rose from 3.417 million in 2018 to 12.279 million doses in 2020, according to data from China’s routine vaccine statement. However, the overall relative HPV vaccination rate is still at a low level. This is likely one of the reasons why HPV-16, 18 and other genotypes remain the main prevalent genotypes in China so far.

In addition, our study shows that HPV-52, 58, 16, 51 may be the key prevalent genotypes in Guangzhou. Among the vaccines currently approved for marketing in China, only the nine-valent vaccine contains these four genotypes. However, the HPV nine-valent vaccine in Guangzhou is currently available in limited number of injections due to its limited quantity and high price. This may be an important reason for the prevalence of HPV-52 genotypes in Guangzhou.

In response to the WHO’s call for global action to eliminate cervical cancer announced in 2018, China has actively strengthened the scientific publicity of HPV vaccination and promoted the pilot work of HPV vaccine. It has initiated the implementation of free domestic bivalent vaccination for girls of the right age across the province in Guangdong, Hainan, and Fujian provinces, incorporating this work into a project of the provincial government to do practical things for the people in 2022. Pilot cities such as Jinan, Xiamen, Wuxi, and Ordos have introduced a policy of free HPV vaccination for school-age girls. Chengdu has given a flat-rate subsidy for HPV vaccination for school-age girls, among others. It is hoped that the implementation of these policies will effectively reduce the number of new cases of cervical cancer in China in the future.

In conclusion, the study only analyzed the prevalence of various genotypes of HPV infection among local women for almost 3 years. Data from this study were limited. We did not perform pathological or cytological analyses of the participants’ cervixes. In addition, only patients admitted to our hospital were statistically analyzed, and some patients were included based on their reason for coming to the outpatient center, suggesting that these results may not be the representation of the general female population in Guangzhou and that the study data were limited and representation of the results of this study.

### Supplementary Information

**Additional file 1: Table 1.** HPV prevalence in different disease.** Table 2.** The number and rate of HPV infections in different age groups for different diseases respectively.** Table 3.** Number of HR-HPV, pHR-HPV and LR-HPV infections in different diseases.** Table 4**. Number and rate of infection for different HPV genotypes, respectively.** Table 5**. Number of cases and prevalence of each genotype in HRHPV, LR-HPV and pHR-HPV.** Table 6**. Prevalence of HPV infection at different ages.** Table 7**. Comparison of the prevalence of HPV infection in the >=56 age group with the prevalence of infection in the other groups, respectively.** Table 8**. Prevalence of HPV-52, HPV-58, HPV-16, HPV-51, and HPV-68 in different age groups, respectively.** Table 9**. Comparison of infection rates in different age groups separately and in the age group greater than or equal to 56 years old.** Table 10**. Ratio of single and multiple infections for different HPV genotypes.** Table 11**. Prevalence of HPV-16, 52, 18 and 58 infections in provinces, municipalities and autonomous regions of China, respectively.

## Data Availability

All needed data are available in manuscript and Additional file 1.
