# Supplementary material for: Distribution patterns of human papillomavirus genotypes among women in Guangzhou, China
Source: Infect Agent Cancer. 2023 Oct 31;18:67. doi: 10.1186/s13027-023-00541-8 (PMC10617049; doi:10.1186/s13027-023-00541-8)
Supplement: Supplementary file 1 — Additional file 1: Table 1. HPV prevalence in different disease. Table 2. The number and rate of HPV infections in different age groups for different diseases respectively. Table 3. Number of HR-HPV, pHR-HPV and LR-HPV infections in different diseases. Table 4. Number and rate of infection for different HPV genotypes, respectively. Table 5. Number of cases and prevalence of each genotype in HRHPV, LR-HPV and pHR-HPV. Table 6. Prevalence of HPV infection at different ages. Table 7. Comparison of the prevalence of HPV infection in the >=56 age group with the prevalence of infection in the other groups, respectively. Table 8. Prevalence of HPV-52, HPV-58, HPV-16, HPV-51, and HPV-68 in different age groups, respectively. Table 9. Comparison of infection rates in different age groups separately and in the age group greater than or equal to 56 years old. Table 10. Ratio of single and multiple infections for different HPV genotypes. Table 11. Prevalence of HPV-16, 52, 18 and 58 infections in provinces, municipalities and autonomous regions of China, respectively. [file 13027_2023_541_MOESM1_ESM.docx]

Table 1. HPV prevalence in different disease.

| Clinical diagnosis | No infection | Infection | Total | Percentage of diseases (%) | Infection rate (%) | Age | HPV genotypes |
| --- | --- | --- | --- | --- | --- | --- | --- |
| CIN 1 | 394 | 1,107 | 1,501 | 5.24% | 3.86% | 38.29±10.550 | 11,16,18,31,33,35,39,42,43,44,45,51,52,53,56,58,59,6,66,68,73,81,82,83 |
| CIN 2 | 439 | 1,109 | 1,548 | 5.41% | 3.87% | 38.24±10.499 | 11,16,18,31,33,35,39,42,43,44,45,51,52,53,56,58,59,6,66,68,73,81,82,83 |
| CIN 3 | 333 | 851 | 1,184 | 4.13% | 2.97% | 38.35±10.01 | 11,16,18,31,33,35,39,42,43,44,45,51,52,53,56,58,59,6,66,68,81,82,83 |
| Genital warts | 6 | 4 | 10 | 0.03% | 0.01% | 35.5±6.005 | 51,52,58,6 |
| Physical infertility | 4,962 | 12 | 4,974 | 17.37% | 0.04% | 37.32±9.495 | 16,39,42,44,52,53,58,68 |
| Vaginitis | 7,715 | 1,053 | 8,768 | 30.61% | 3.68% | 36.45±10.848 | 11,16,18,31,33,35,39,42,43,44,45,51,52,53,56,58,59,6,66,68,73,81,82,83 |
| Cervicitis | 9,126 | 1,532 | 10,658 | 37.21% | 5.35% | 36.92±9.329 | 11,16,18,31,33,35,39,42,43,44,45,51,52,53,56,58,59,6,66,68,73,81,82,83 |

CIN: Cervical intraepithelial neoplasia.

Table 2. The number and rate of HPV infections in different age groups for different diseases respectively.

| **Clinical diagnosis**  **(Total number)** | **<=25**  **Number**  **(Ratio)** | **26-30**  **Number**  **(Ratio)** | **31-35**  **Number**  **(Ratio)** | **36-40**  **Number**  **(Ratio)** | **41-45**  **Number**  **(Ratio)** | **46-50**  **Number**  **(Ratio)** | **51-55**  **Number**  **(Ratio)** | **>=56**  **Number**  **(Ratio)** |
| --- | --- | --- | --- | --- | --- | --- | --- | --- |
| CIN 1  (1,501) | 105  (7.00%) | 197  (13.12%) | 231  (15.39%) | 199  (13.26%) | 21  (1.40%) | 99  (6.60%) | 73  (4.86%) | 82  (5.46%) |
| CIN 2  (1,548) | 100  (6.46%) | 204  (13.18%) | 263  (16.99%) | 161  (10.40%) | 130  (8.40%) | 93  (6.01%) | 59  (3.81%) | 99  (6.40%) |
| CIN 3  (1,184) | 70  (5.91%) | 136  (11.49%) | 213  (17.99%) | 155  (13.09%) | 96  (8.11%) | 72  (6.08%) | 46  (3.89%) | 63  (5.32%) |
| Genital warts  (10) | 0  (0.00) | 1  (10.00%) | 0  (0.00) | 2  (20.00%) | 0  (0.00%) | 1  (10.00%) | 0  (0.00) | 0  (0.00) |
| Physical infertility  (4,974) | 2  (0.04%) | 3  (0.06%) | 6  (0.12%) | 1  (0.02%) | 0  (0.00) | 0  (0.00) | 0  (0.00) | 0  (0.00) |
| Vaginitis  (8,768) | 114  (1.30%) | 218  (2.49%) | 277  (3.16%) | 175  (2.00%) | 84  (0.96%) | 70  (0.80%) | 56  (0.64%) | 59  (0.67%) |
| Cervicitis  (10,658) | 156  (1.46%) | 326  (3.06%) | 353  (3.31%) | 239  (2.24%) | 155  (1.45%) | 112  (1.05%) | 80  (0.75%) | 211  (1.98%) |

Table 3. Number of HR-HPV, pHR-HPV and LR-HPV infections in different diseases.

| Clinical diagnosis | HR-HPV | pHR-HPV | LR-HPV |
| --- | --- | --- | --- |
| CIN 1 | 779 | 188 | 206 |
| CIN 2 | 792 | 192 | 198 |
| CIN 3 | 596 | 169 | 142 |
| Genital warts | 3 | 0 | 1 |
| Physical infertility | 7 | 3 | 3 |
| Vaginitis | 754 | 177 | 185 |
| Cervicitis | 1,061 | 276 | 286 |

Table 4. Number and rate of infection for different HPV genotypes, respectively.

| HPV genotype | Number of cases | Infection rate (%) |
| --- | --- | --- |
| 52 | 1,432 | 4.99 |
| 58 | 625 | 2.18 |
| 16 | 608 | 2.12 |
| 51 | 462 | 1.61 |
| 68 | 370 | 1.29 |
| 53 | 349 | 1.21 |
| 39 | 342 | 1.19 |
| 56 | 314 | 1.09 |
| 42 | 312 | 1.08 |
| 59 | 244 | 0.85 |
| 44 | 222 | 0.77 |
| 66 | 221 | 0.77 |
| 18 | 207 | 0.72 |
| 81 | 195 | 0.68 |
| 33 | 177 | 0.61 |
| 31 | 153 | 0.53 |
| 6 | 138 | 0.48 |
| 43 | 116 | 0.40 |
| 82 | 73 | 0.25 |
| 11 | 68 | 0.23 |
| 35 | 60 | 0.20 |
| 45 | 49 | 0.17 |
| 73 | 48 | 0.16 |
| 83 | 21 | 0.07 |

Table 5. Number of cases and prevalence of each genotype in HR-HPV, LR-HPV and pHR-HPV.

| HR-HPV genotype | Number of cases | Percentage (%) (3,992) |
| --- | --- | --- |
| 52 | 1,432 | 35.87 |
| 58 | 625 | 15.66 |
| 16 | 608 | 15.23 |
| 51 | 462 | 11.57 |
| 39 | 342 | 8.57 |
| 56 | 314 | 7.87 |
| 59 | 244 | 6.11 |
| 18 | 207 | 5.19 |
| 33 | 177 | 4.43 |
| 31 | 153 | 3.83 |
| 35 | 60 | 1.50 |
| 45 | 49 | 1.23 |

| pHR-HPV genotype | Number of cases | Percentage (%) (1,005) |
| --- | --- | --- |
| 68 | 370 | 36.82 |
| 53 | 349 | 34.73 |
| 66 | 221 | 21.99 |
| 82 | 73 | 7.26 |
| 73 | 48 | 4.78 |

| LR-HPV genotype | Number of cases | Percentage (%) (1,021) |
| --- | --- | --- |
| 42 | 312 | 30.56 |
| 44 | 222 | 21.74 |
| 81 | 195 | 19.10 |
| 6 | 138 | 13.52 |
| 43 | 116 | 11.36 |
| 11 | 70 | 6.86 |
| 83 | 21 | 2.06 |

Table 6. Prevalence of HPV infection at different ages.

| **Age groups** | **Average of ages**  **（X̅±SD）** | **Total number** | **Total infection**  **number**  **(rate%)** | **HR-HPV number**  **(rate%)** | **pHR-HPV number**  **(rate%)** | **LR-HPV**  **number**  **(rate%)** |
| --- | --- | --- | --- | --- | --- | --- |
| **<=25** | 23.35±1.80 | 2,007 | 482 (24.02%) | 297(14.08%) | 102(5.08%) | 83(4.14%) |
| 26~30 | 28.33±1.35 | 5,742 | 1,109(19.31%) | 804(14.00%) | 188(3.27%) | 184(3.20%) |
| 31~35 | 32.92±1.43 | 7,082 | 1,343(18.96%) | 937(13.23%) | 231(3.26%) | 250(3.53%) |
| 36~40 | 37.76±1.38 | 5,278 | 989(18.74%) | 687(13.02%) | 165(3.13%) | 187(3.54%) |
| 41~45 | 42.88±1.46 | 3,160 | 603(19.08%) | 418(13.23%) | 113(3.58%) | 107(3.39%) |
| 46~50 | 47.87±1.41 | 2,487 | 459(18.46%) | 325(13.07%) | 73(2.94%) | 90(3.62%) |
| 51~55 | 52.67±1.39 | 1,461 | 304(20.81%) | 211(14.44%) | 45(3.08%) | 51(3.49%) |
| >=56 | 61.07±4.86 | 1,426 | 379(26.58%) | 267(18.72%) | 62(4.35%) | 70(4.91%) |

Table 7. Comparison of the prevalence of HPV infection in the >=56 age group with the prevalence of infection in the other groups, respectively.

<=25 VS >=56 a * b Crosstabulation

|  | | | b | | Total |
| --- | --- | --- | --- | --- | --- |
|  |  |  | Infection | No infection |  |
| a | <=25 | Count | 482 | 1525 | 2007 |
|  |  | Expected Count | 503.4 | 1503.6 | 2007.0 |
|  |  | % within a | 24.0% | 76.0% | 100.0% |
|  | >=56 | Count | 379 | 1047 | 1426 |
|  |  | Expected Count | 357.6 | 1068.4 | 1426.0 |
|  |  | % within a | 26.6% | 73.4% | 100.0% |
| Total |  | Count | 861 | 2572 | 3433 |
|  |  | Expected Count | 861.0 | 2572.0 | 3433.0 |
|  |  | % within a | 25.1% | 74.9% | 100.0% |

|  | Value | df | Asymp. Sig.  (2-sided) | Exact Sig.  (2-sided) | | Exact Sig.  (1-sided) | Point Probability |
| --- | --- | --- | --- | --- | --- | --- | --- |
| Pearson Chi-Square | 2.912^a^ | 1 | .088 | .093 | .048 | |  |
| Continuity Correction^b^ | 2.777 | 1 | .096 |  |  | |  |
| Likelihood Ratio | 2.902 | 1 | .088 | .093 | .048 | |  |
| Fisher's Exact Test |  |  |  | .093 | .048 | |  |
| Linear-by-Linear Association | 2.911^c^ | 1 | .088 | .093 | .048 | | .007 |
| N of Valid Cases | 3433 |  |  |  |  | |  |

26-30 VS >=56 a * b Crosstabulation

|  | | | b | | | Total |
| --- | --- | --- | --- | --- | --- | --- |
|  |  |  | Infection | No infection | |  |
| a | 26-30 | Count | 1109 | 4633 | 5742 | |
|  |  | Expected Count | 1192.0 | 4550.0 | 5742.0 | |
|  |  | % within a | 19.3% | 80.7% | 100.0% | |
|  | >=56 | Count | 379 | 1047 | 1426 | |
|  |  | Expected Count | 296.0 | 1130.0 | 1426.0 | |
|  |  | % within a | 26.6% | 73.4% | 100.0% | |
| Total |  | Count | 1488 | 5680 | 7168 | |
|  |  | Expected Count | 1488.0 | 5680.0 | 7168.0 | |
|  |  | % within a | 20.8% | 79.2% | 100.0% | |

|  | Value | df | Asymp. Sig. (2-sided) | Exact Sig. (2-sided) | Exact Sig. (1-sided) | Point Probability |
| --- | --- | --- | --- | --- | --- | --- |
| Pearson Chi-Square | 36.642^a^ | 1 | .000 | .000 | .000 |  |
| Continuity Correction^b^ | 36.202 | 1 | .000 |  |  |  |
| Likelihood Ratio | 35.015 | 1 | .000 | .000 | .000 |  |
| Fisher's Exact Test |  |  |  | .000 | .000 |  |
| Linear-by-Linear Association | 36.637^c^ | 1 | .000 | .000 | .000 | .000 |
| N of Valid Cases | 7168 |  |  |  |  |  |

31-35 VS >=56 a * b Crosstabulation

|  | | | | b | | Total |
| --- | --- | --- | --- | --- | --- | --- |
|  |  |  |  | Infection | No infection |  |
| a | 31-35 | | Count | 1343 | 5739 | 7082 |
|  |  | | Expected Count | 1433.4 | 5648.6 | 7082.0 |
|  |  | | % within a | 19.0% | 81.0% | 100.0% |
|  | >=56 | | Count | 379 | 1047 | 1426 |
|  | |  | Expected Count | 288.6 | 1137.4 | 1426.0 |
|  | |  | % within a | 26.6% | 73.4% | 100.0% |
| Total | |  | Count | 1722 | 6786 | 8508 |
|  | |  | Expected Count | 1722.0 | 6786.0 | 8508.0 |
|  | |  | % within a | 20.2% | 79.8% | 100.0% |

|  | Value | df | Asymp. Sig. (2-sided) | Exact Sig. (2-sided) | Exact Sig. (1-sided) | Point Probability |
| --- | --- | --- | --- | --- | --- | --- |
| Pearson Chi-Square | 42.630^a^ | 1 | .000 | .000 | .000 |  |
| Continuity Correction^b^ | 42.159 | 1 | .000 |  |  |  |
| Likelihood Ratio | 40.380 | 1 | .000 | .000 | .000 |  |
| Fisher's Exact Test |  |  |  | .000 | .000 |  |
| Linear-by-Linear Association | 42.625^c^ | 1 | .000 | .000 | .000 | .000 |
| N of Valid Cases | 8508 |  |  |  |  |  |

36-40 VS >=56 a * b Crosstabulation

|  | | | b | | Total |
| --- | --- | --- | --- | --- | --- |
|  |  |  | Infection | No infection |  |
| a | 36-40 | Count | 989 | 4289 | 5278 |
|  |  | Expected Count | 1077.0 | 4201.0 | 5278.0 |
|  |  | % within a | 18.7% | 81.3% | 100.0% |
|  | >=56 | Count | 379 | 1047 | 1426 |
|  |  | Expected Count | 291.0 | 1135.0 | 1426.0 |
|  |  | % within a | 26.6% | 73.4% | 100.0% |
| Total |  | Count | 1368 | 5336 | 6704 |
|  |  | Expected Count | 1368.0 | 5336.0 | 6704.0 |
|  |  | % within a | 20.4% | 79.6% | 100.0% |

|  | Value | df | Asymp. Sig. (2-sided) | Exact Sig. (2-sided) | Exact Sig. (1-sided) | Point Probability |
| --- | --- | --- | --- | --- | --- | --- |
| Pearson Chi-Square | 42.483^a^ | 1 | .000 | .000 | .000 |  |
| Continuity Correction^b^ | 42.002 | 1 | .000 |  |  |  |
| Likelihood Ratio | 40.519 | 1 | .000 | .000 | .000 |  |
| Fisher's Exact Test |  |  |  | .000 | .000 |  |
| Linear-by-Linear Association | 42.477^c^ | 1 | .000 | .000 | .000 | .000 |
| N of Valid Cases | 6704 |  |  |  |  |  |

41-45 VS >=56 a * b Crosstabulation

|  | | | b | | Total |
| --- | --- | --- | --- | --- | --- |
|  |  |  | Infection | No infection |  |
| a | 41-45 | Count | 603 | 2557 | 3160 |
|  |  | Expected Count | 676.7 | 2483.3 | 3160.0 |
|  |  | % within a | 19.1% | 80.9% | 100.0% |
|  | >=56 | Count | 379 | 1047 | 1426 |
|  |  | Expected Count | 305.3 | 1120.7 | 1426.0 |
|  |  | % within a | 26.6% | 73.4% | 100.0% |
| Total |  | Count | 982 | 3604 | 4586 |
|  |  | Expected Count | 982.0 | 3604.0 | 4586.0 |
|  |  | % within a | 21.4% | 78.6% | 100.0% |

|  | Value | df | Asymp. Sig. (2-sided) | Exact Sig. (2-sided) | Exact Sig. (1-sided) | Point Probability |
| --- | --- | --- | --- | --- | --- | --- |
| Pearson Chi-Square | 32.806^a^ | 1 | .000 | .000 | .000 |  |
| Continuity Correction^b^ | 32.362 | 1 | .000 |  |  |  |
| Likelihood Ratio | 31.925 | 1 | .000 | .000 | .000 |  |
| Fisher's Exact Test |  |  |  | .000 | .000 |  |
| Linear-by-Linear Association | 32.799^c^ | 1 | .000 | .000 | .000 | .000 |
| N of Valid Cases | 4586 |  |  |  |  |  |

46-50 VS >=56 a * b Crosstabulation

|  | | | b | | Total |
| --- | --- | --- | --- | --- | --- |
|  |  |  | Infection | No infection |  |
| a | 46-50 | Count | 459 | 2028 | 2487 |
|  |  | Expected Count | 532.6 | 1954.4 | 2487.0 |
|  |  | % within a | 18.5% | 81.5% | 100.0% |
|  | >=56 | Count | 379 | 1047 | 1426 |
|  |  | Expected Count | 305.4 | 1120.6 | 1426.0 |
|  |  | % within a | 26.6% | 73.4% | 100.0% |
| Total |  | Count | 838 | 3075 | 3913 |
|  |  | Expected Count | 838.0 | 3075.0 | 3913.0 |
|  |  | % within a | 21.4% | 78.6% | 100.0% |

|  | Value | df | Asymp. Sig. (2-sided) | Exact Sig. (2-sided) | Exact Sig. (1-sided) | Point Probability |
| --- | --- | --- | --- | --- | --- | --- |
| Pearson Chi-Square | 35.525^a^ | 1 | .000 | .000 | .000 |  |
| Continuity Correction^b^ | 35.044 | 1 | .000 |  |  |  |
| Likelihood Ratio | 34.829 | 1 | .000 | .000 | .000 |  |
| Fisher's Exact Test |  |  |  | .000 | .000 |  |
| Linear-by-Linear Association | 35.515^c^ | 1 | .000 | .000 | .000 | .000 |
| N of Valid Cases | 3913 |  |  |  |  |  |

51-55 VS >=56 a * b Crosstabulation

|  | | | b | | Total |
| --- | --- | --- | --- | --- | --- |
|  |  |  | Infection | No infection |  |
| a | 51-55 | Count | 304 | 1157 | 1461 |
|  |  | Expected Count | 345.6 | 1115.4 | 1461.0 |
|  |  | % within a | 20.8% | 79.2% | 100.0% |
|  | >=56 | Count | 379 | 1047 | 1426 |
|  |  | Expected Count | 337.4 | 1088.6 | 1426.0 |
|  |  | % within a | 26.6% | 73.4% | 100.0% |
| Total |  | Count | 683 | 2204 | 2887 |
|  |  | Expected Count | 683.0 | 2204.0 | 2887.0 |
|  |  | % within a | 23.7% | 76.3% | 100.0% |

|  | Value | df | Asymp. Sig. (2-sided) | Exact Sig. (2-sided) | Exact Sig. (1-sided) | Point Probability |
| --- | --- | --- | --- | --- | --- | --- |
| Pearson Chi-Square | 13.303^a^ | 1 | .000 | .000 | .000 |  |
| Continuity Correction^b^ | 12.986 | 1 | .000 |  |  |  |
| Likelihood Ratio | 13.320 | 1 | .000 | .000 | .000 |  |
| Fisher's Exact Test |  |  |  | .000 | .000 |  |
| Linear-by-Linear Association | 13.299^c^ | 1 | .000 | .000 | .000 | .000 |
| N of Valid Cases | 2887 |  |  |  |  |  |

Table 8. Prevalence of HPV-52, HPV-58, HPV-16, HPV-51, and HPV-68 in different age groups, respectively.

| HPV genotype | <=25 | 26-30 | 31-35 | 36-40 | 41-45 | 46-50 | 51-55 | >=56 |
| --- | --- | --- | --- | --- | --- | --- | --- | --- |
| HPV52 | 6.33% | 5.19% | 4.22% | 4.83% | 4.81% | 4.74% | 5.34% | 7.36% |
| HPV58 | 3.14% | 2.30% | 2.13% | 1.76% | 2.25% | 1.85% | 2.60% | 2.17% |
| HPV16 | 2.64% | 2.18% | 2.16% | 1.82% | 1.77% | 2.01% | 2.67% | 2.52% |
| HPV51 | 2.59% | 1.57% | 1.75% | 1.14% | 1.30% | 1.73% | 1.16% | 2.45% |
| HPV68 | 1.74% | 1.29% | 1.14% | 1.27% | 1.39% | 1.13% | 1.37% | 1.47% |

Table 9. Comparison of infection rates in different age groups separately and in the age group greater than or equal to 56 years old.

| **Age group** | ***P*-value** |
| --- | --- |
| <=25 VS >=56 | 0.234 |
| 26~30 VS >=56 | 0.001 |
| 31~35 VS >=56 | 0 |
| 36~40 VS >=56 | 0 |
| 41~45 VS >=56 | 0.001 |
| 46~50 VS >=56 | 0.001 |
| 51~55 VS >=56 | 0.026 |

Table 10. Ratio of single and multiple infections for different HPV genotypes.

| HR-HPV genotype | Total infection | Single infection rates (%) (5,668) | Multiple infection rates (%) (5,668) |
| --- | --- | --- | --- |
| 52 | 1,432 | 18.86 | 6.40 |
| 58 | 625 | 7.48 | 3.55 |
| 16 | 608 | 7.32 | 3.41 |
| 51 | 462 | 5.13 | 3.02 |
| 39 | 342 | 3.92 | 2.12 |
| 56 | 314 | 3.48 | 2.06 |
| 59 | 244 | 2.10 | 2.21 |
| 18 | 207 | 2.54 | 1.11 |
| 33 | 177 | 1.94 | 1.18 |
| 31 | 153 | 1.38 | 1.32 |
| 35 | 60 | 0.62 | 0.44 |
| 45 | 49 | 0.60 | 0.26 |

| pHR-HPV genotype | Total infection | Single infection rates (%) (5,668) | Multiple infection rates (%) (5,668) |
| --- | --- | --- | --- |
| 68 | 370 | 4.00 | 2.52 |
| 53 | 349 | 3.90 | 2.26 |
| 66 | 221 | 2.38 | 1.52 |
| 82 | 73 | 0.62 | 0.67 |
| 73 | 48 | 0.44 | 0.41 |

| LR-HPV genotype | Total infection | Single infection rates(%)(5668) | Multiple infection rates(%)(5668) |
| --- | --- | --- | --- |
| 42 | 312 | 4.98 | 0.53 |
| 44 | 222 | 3.56 | 0.35 |
| 81 | 195 | 3.16 | 0.28 |
| 6 | 138 | 2.13 | 0.30 |
| 43 | 116 | 1.80 | 0.25 |
| 11 | 70 | 1.06 | 0.18 |
| 83 | 21 | 0.23 | 0.14 |

Table 11. Prevalence of HPV-16, 52, 18 and 58 infections in provinces, municipalities and autonomous regions of China, respectively.

| District | HPV-16 prevalence(%) | HPV-52 prevalence(%) | HPV-18 prevalence(%) | HPV-58 prevalence(%) | reference |
| --- | --- | --- | --- | --- | --- |
| Beijin | 3.3 | 6.2 | 1.4 | 4.7 | ^1^ |
| Tianjing | 11.77 | 12.79 | 4.61 | 15.05 | ^2^ |
| Hebei | 17.33 | 10.36 | 4.4 | 11.15 | ^3^ |
| Shanxi | 6.09 | 3.59 | 2.04 | 4.08 | ^4^ |
| Inner Mongolia | 19.98 | 16.15 | 7.76 | 11.77 | ^5^ |
| Liaoning | 26.2 | 19.4 | 7.5 | 13.8 | ^6^ |
| Jilin | 12 | 5.5 | 3.2 | 6.1 | ^7^ |
| Heilongjiang | 11.8 | 5.8 | 4.2 | 8 | ^7^ |
| Shanghai | 1.94 | 2.78 | 0.86 | 1.84 | ^8^ |
| Jiangsu | 12.63 | 20.93 | 2.62 | 14.43 | ^9^ |
| Zhejiang | 2.5 | 3.1 | 0.8 | 2.1 | ^10^ |
| Anhui | 16.84 | 20.07 | 8.26 | 6.94 | ^11^ |
| Fujian | 1.23 | 2.54 | 0.73 | 1.31 | ^12^ |
| Jiangxi | 2.63 | 5.38 | 0.95 | 1.79 | ^13^ |
| Shandong | 3.34 | 2.5 | 1.13 | 2.62 | ^14^ |
| Henan | 16.73 | 9.11 | 0.61 | 10.17 | ^15^ |
| Hubei | 2.56 | 4.23 | 1.02 | 2.37 | ^16^ |
| Hunan | 4.7 | 4.5 | 1.2 | 3.1 | ^17^ |
| Guangdong | 2.98 | 4.16 | 1.1 | 2.15 | ^18^ |
| Guangxi | 4.54 | 5.07 | 1.49 | 3.24 | ^19^ |
| Hainan | 11.08 | 20.4 | 4.72 | 11.2 | ^20^ |
| Chongqing | 2.09 | 3.2 | 0.58 | 1.94 | ^21^ |
| Sichuan | 13.6 | 21.05 | 4.97 | 15.14 | ^22^ |
| Guizhou | 15.9 | 7.2 | 5.8 | 5.8 | ^23^ |
| Yunnan | 13.48 | 17.9 | 3.91 | 11.59 | ^24^ |
| Tibet | 14.5 | 24.8 | 3.4 | 14.5 | ^25^ |
| Shananxi | 6.3 | 6.09 | 2.05 | 3.77 | ^26^ |
| Gansu | 28.59 | 13.34 | 7.77 | 17.01 | ^27^ |
| Qinghai | 22.21 | 8.55 | 4.7 | 13.74 | ^28^ |
| Ningxia | 10.8 | 4 | 0.6 | 4.2 | ^29^ |
| Xinjiang | 5.18 | 0.89 | 0.67 | 0.94 | ^30^ |
| Taiwan | 4.21 | 3.65 | 1.99 | 3.01 | ^31^ |
| Hong Kong | 1.24 | 1.16 | 0.99 | 1.03 | ^32^ |
| Macau | 15.5 | 26.8 | 8.9 | 8.9 | ^33^ |

**Reference**

1. Zhu X, Wang Y, Lv Z, Su J. Prevalence and genotype distribution of high-risk HPV infection among women in Beijing, China. *J Med Virol*. 2021;93:5103-5109. doi: 10.1002/jmv.27013

2. 张春玲, 周大兵, 张晶. 天津滨海新区3447例妇女宫颈HPV感染型别分析. *医学理论与实践*. 2019;32:2100-2101. doi: 10.19381/j.issn.1001-7585.2019.13.072

3. 徐帅师, 牛凤霞, 高瑾, 张咏梅. 河北地区26385例女性宫颈细胞HPV基因分型分析. *中国微生态学杂志*. 2018;30:947-950. doi: 10.13381/j.cnki.cjm.201808018

4. 田凌君. 12691例山西大医院妇产科门诊就诊患者HPV感染的调查研究. In: 山西医科大学; 2017.

5. 贺晓花, 田晓燕, 王振飞, 陈永霞, 牟永平. 内蒙古地区人乳头状瘤病毒感染基因亚型分析. *中国卫生检验杂志*. 2020;30:1698-1700.

6. Xue H, Lin X, Li T, Yan X, Guo K, Zhang Y. Prevalence and genotype distribution of human papillomavirus infection in asymptomatic women in Liaoning province, China. *J Med Virol*. 2015;87:1248-1253. doi: 10.1002/jmv.24029

7. 娄咏崴, 谷振环. 吉林地区、黑龙江地区人乳头瘤病毒感染状况分析. *中国实验诊断学*. 2012;16:2279-2280.

8. Li X, Xiang F, Dai J, Zhang T, Chen Z, Zhang M, Wu R, Kang X. Prevalence of cervicovaginal human papillomavirus infection and genotype distribution in Shanghai, China. *Virol J*. 2022;19:146. doi: 10.1186/s12985-022-01879-y

9. Wang L, Chen G, Jiang J. Genotype Distribution and Prevalence of Human Papillomavirus Infection in Women in Northern Jiangsu Province of China. *Cancer Manag Res*. 2021;13:7365-7372. doi: 10.2147/CMAR.S332769

10. 叶菁. 浙江地区HPV流行病学调查及宫颈癌危险因素在HPV筛查中的作用. In: 浙江大学; 2010.

11. 余娟平, 魏琦, 王倩倩, 常中宝, 叶红, 徐庆华, 李晓华. 安徽地区17160例健康体检女性人乳头瘤病毒感染状况及基因分型. *中华实验和临床感染病杂志(电子版)*. 2019;13:389-395.

12. 吴凯. 福建地区女性HPV感染的流行病学特征与宫颈早期病变的关系. *临床合理用药杂志*. 2019;12:165-167. doi: 10.15887/j.cnki.13-1389/r.2019.22.094

13. 吴昌志, 林梦麒. 2021年江西地区138392例女性HPV感染亚型分析. *中国预防医学杂志*.1-6.

14. 杨艳红. 山东地区女性健康体检时HPV病毒感染的检出情况研究. *河北医药*. 2019;41:3803-3806.

15. 智艳芳, 李肖甫, 班振英, 邱翠, 荣守华, 张新. 河南地区女性HPV感染及基因型分布研究. *中华肿瘤防治杂志*. 2014;21:660-664. doi: 10.16073/j.cnki.cjcpt.2014.09.001

16. Xiang F, Guan Q, Liu X, Xiao H, Xia Q, Liu X, Sun H, Song X, Zhong Y, Yuan CH, et al. Distribution characteristics of different human papillomavirus genotypes in women in Wuhan, China. *J Clin Lab Anal*. 2018;32:e22581. doi: 10.1002/jcla.22581

17. 王丹琳. 湖南地区宫颈人乳头状瘤病毒感染情况的Meta分析. In: 南华大学; 2019.

18. Huang W, Xu H, Hu H, Zhang D, Liu Y, Guo Y, Xiao F, Chen W, Ma Z. The prevalence of human papillomavirus among women in northern Guangdong Province of China. *Sci Rep*. 2022;12:13353. doi: 10.1038/s41598-022-17632-y

19. 韦晓宁. 第一部分 广西地区女性生殖道人乳头状瘤病毒感染的流行病学特征研究 第二部分 人脐带间充质干细胞条件培养基对人子宫内膜上皮细胞的促增殖作用并对Wnt/β-catenin信号通路的影响研究. In: 北京协和医学院; 2022.

20. 陈春宝, 苏震, 田昕, 黄芳, 王洁. 海南2939例女性HPV感染现状及基因型分布. *中国热带医学*. 2017;17:904-907. doi: 10.13604/j.cnki.46-1064/r.2017.09.13

21. 吕静, 廖丹, 陈敏, 种慧敏, 唱凯, 皮燕. 重庆地区女性体检人群高危型人乳头瘤病毒基因亚型分布特征研究. *国际检验医学杂志*. 2020;41:2112-2115+2120.

22. Luo Q, Jiang N, Wu Q, Wang J, Zhong J. Prevalence and genotype distribution of HPV and cervical pathological results in Sichuan Province, China: a three years surveys prior to mass HPV vaccination. *Virol J*. 2020;17:100. doi: 10.1186/s12985-020-01366-2

23. 高超, 赵丹青, 孙磊, 朱科静, 许永劼, 朱金凤, 金玉姬, 潘卫. 505例女性体检人群人乳头状瘤病毒感染情况及基因亚型分布. *贵州医科大学学报*. 2019;44:1330-1333+1359. doi: 10.19367/j.cnki.1000-2707.2019.11.017

24. 曹杰贤, 李雪菲, 沈国玲, 楚青. 云南7个地区女性HPV亚型感染情况调查. *检验医学与临床*. 2021;18:789-792.

25. 普布卓玛, 边巴卓玛, 徐小红, 尼玛卓嘎, 普布央宗, 普布德吉, 次仁拉姆, 索朗卡珠, 扎西曲宗, 小尼玛卓嘎, et al. 西藏高海拔地区妊娠期妇女宫颈HPV感染状况及相关因素分析. *中国生育健康杂志*. 2020;31:522-525.

26. 王蕾, 何小波, 周再铜, 赵芳平, 王敏, 刘杨, 刘孟黎. 陕西地区9749例女性HPV感染情况及基因分型研究. *海南医学*. 2019;30:3200-3203.

27. 杜宏, 索兰草, 刘红贤, 王强, 刘斌, 简启亮, 黄山, 张文华, 王芳. 甘肃地区女性宫颈HPV感染的现状研究. *暨南大学学报(自然科学与医学版)*. 2015;36:40-45.

28. 靳冬梅. *青海地区46237例门诊女性宫颈HPV检测结果分析*.

29. 牛萌, 肖冬梅, 刘国莲, 何旭文, 陈红, 刘赟赟. 宁夏部分女性对宫颈癌及其疫苗的认知情况与接种意愿调查分析. *中外女性健康研究*. 2019:19-21+31.

30. 李海涛, 朱琳, 董璇, 王岩. 中国新疆地区宫颈人乳头瘤病毒感染情况的Meta分析. *中国妇幼健康研究*. 2022;33:54-61.

31. Wang CC, Chang SL, Chu FY, Cheng CY, Cheng SH. Human papillomavirus infection and anal cytology in Taiwanese homosexual men with and without HIV infection. *J Infect Dev Ctries*. 2019;13:318-325. doi: 10.3855/jidc.11162

32. Liu SS, Chan KY, Leung RC, Chan KK, Tam KF, Luk MH, Lo SS, Fong DY, Cheung AN, Lin ZQ, et al. Prevalence and risk factors of Human Papillomavirus (HPV) infection in southern Chinese women - a population-based study. *PLoS One*. 2011;6:e19244. doi: 10.1371/journal.pone.0019244

33. Yip YC, Ngai KL, Vong HT, Tzang LC, Ji S, Yang M, Chan PK. Prevalence and genotype distribution of cervical human papillomavirus infection in Macao. *J Med Virol*. 2010;82:1724-1729. doi: 10.1002/jmv.21826
